# Understanding What Emergency Medicine (EM) Faculty Want: Preferences for Content and Delivery of Faculty-Focused Departmental Education

**DOI:** 10.7759/cureus.82787

**Published:** 2025-04-22

**Authors:** Justin G Myers, Aalap Shah, Neeraja Murali, Christopher Reilly, Joshua Glasser, Christina Shenvi

**Affiliations:** 1 Emergency Medicine, University of North Carolina, Chapel Hill School of Medicine, Chapel Hill, USA; 2 Emergency Medicine, Medical University of South Carolina, Charleston, USA; 3 Emergency Medicine, University of Maryland School of Medicine, Baltimore, USA; 4 Emergency Medicine, Maimonides Medical Center, Brooklyn, USA; 5 Emergency Medicine, Penn State University College of Medicine, Milton S. Hershey Medical Center, Hershey, USA

**Keywords:** continuing medical education (cme), emergency medicine physician, faculty development programs, grand rounds, utilizing group learning

## Abstract

Background

Methods for ongoing faculty education in academic emergency medicine (EM) have evolved over the years. In addition to traditional in-person lectures or grand rounds, education may be supplemented by online or asynchronous content. The preferences of EM physicians for faculty education content and format are not well understood. We sought to determine barriers and facilitating factors for EM faculty to participate in educational sessions and/or content, as well as faculty preferences on subject matter, content delivery methods, and personnel.

Methods

We performed a cross-sectional survey of EM faculty physicians from 18 US academic medical centers. Survey invitations were delivered by a site representative at each institution using a Qualtrics survey instrument (Qualtrics, Provo, US). Quantitative data were analyzed with descriptive statistics via percentiles. Qualitative data were analyzed using thematic analysis.

Results

A total of 230 individuals from 18 academic departments across the US responded to the survey. Clinical experience ranged from 0-5 years (25%) to 21+ years (20%). Lectures were the most preferred format, cited by 90% of participants, followed by hands-on sessions (66%), interactive sessions (47%), and journal clubs (46%). A hybrid format (virtual + in-person) was the most desired delivery format as well as the most common delivery method. Faculty considered clinical duties as the greatest barrier to attending grand rounds (55%), followed by conflicts with appointments/meetings (51%) and uninteresting topics or content (17%). Faculty preferred more content on procedural skills or hands-on training, critical care/resuscitation, and academic development/teaching techniques.

Conclusions

Faculty development opportunities are important for physicians to maintain and grow their skills and knowledge. However, there are many barriers and competing priorities to attendance. Faculty were most motivated to attend based on the topic and the speaker’s skill and reputation. They also valued hybrid and asynchronous options to allow attendance around other clinical and academic duties. Organizers of grand rounds sessions should consider their faculty’s particular needs, interests, and motivations to plan more valuable programming and maximize engagement.

## Introduction

Emergency medicine (EM) department-sponsored, faculty-focused education is changing rapidly. Traditionally, department-sponsored education was termed “grand rounds” and consisted of an in-person lecture delivered in an auditorium [1]. The advancement of adult pedagogy has shifted the sole reliance on medical lecture-based material [2] to other effective teaching methods, such as skill-based simulation [3]. In addition, following the COVID-19 pandemic, the format of delivery has changed, with many institutions offering virtual, hybrid, or recorded sessions. In-person sessions also limit participation in healthcare systems that have geographically dispersed facilities and physicians/staff. The contemporary definition of “grand rounds” could be considered any regularly offered department-sponsored faculty education [4,5]. 

Although there are an abundance of continuing medical education opportunities for physicians, department-sponsored faculty education can provide unique benefits. These benefits include convenient continuing medical education (CME) hours, promotion of collegiality, interprofessional collaboration, resident learner participation, linkage of hospital care systems, departmental research updates, consistency of practice and knowledge across different emergency departments (EDs) within a system, and alignment of department practices [4,6] with evidence. The goals for this education may vary by institution. As departmental leaders plan their faculty education programs, they should consider their desired outcomes as well as faculty preferences and interests.

There is minimal research on the preferences of EM physicians concerning faculty education. Academic medical literature does suggest various improvements for contemporary grand rounds, but the unique schedules, interests, and practice patterns in EM must be considered when implementing changes. Preferences for the mode of delivery of CME for EM faculty have been studied [7], but were not specific to department-based education. Similarly, motivation for attending resident-specific but not faculty-focused educational sessions has been evaluated [8]. Furthermore, faculty preferences have likely changed since the COVID-19 pandemic.

During the pandemic, there was an increase in online, synchronous, asynchronous, and hybrid delivery of faculty education [9]. In-person meetings decreased significantly, with multiple studies assessing this experience among EM faculty and residents [10-12], as well as the cost-effectiveness of virtual grand rounds [13]. Since COVID-19, EM residency programs have returned to an in-person format, with a majority also utilizing a hybrid model [14]. However, now that in-person meetings have resumed to varying degrees, the current educational preferences of EM faculty remain unknown.

We sought to discover EM faculty preferences, needs, and interests related to faculty education. The specific objectives of this study were to determine what motivates EM faculty to attend departmental grand rounds (faculty education) sessions, the barriers they face to attending, the best delivery methods, and the preferred content and speaker types.

## Materials and methods

We conducted a multi-institutional, cross-sectional, survey-based study aimed at understanding the experiences and perspectives of EM faculty physicians from 18 academic medical centers across the US. The study was designed to gather comprehensive data through a structured survey distributed via a Qualtrics platform (Qualtrics, Provo, US). Invitations to participate in the survey were sent by faculty site representatives at each of the 18 institutions. These representatives were part of a network of EM faculty, all of whom were alumni of the American College of Emergency Physicians' Teaching Fellowship [15]. This network of experienced physicians collectively formed the EM Faculty Education Research Team, which played a pivotal role in facilitating the study across multiple institutions.

The survey included both quantitative and qualitative data components. For the quantitative data, descriptive statistics, including percentiles and Likert scale responses, were employed to provide a detailed analysis of trends and patterns across various aspects of EM faculty development. Qualitative data were analyzed through descriptive analysis, allowing for an exploration of the nuanced experiences and insights of the faculty members. This combination of methodologies ensured a robust and comprehensive understanding of the subject matter.

Participation in the study was voluntary, and all responses were anonymous. No identifying information was collected from the participants, preserving their privacy throughout the process. The study was reviewed and deemed Institutional Review Board (IRB)-exempt by the IRB of the University of North Carolina (UNC) under protocol number #24-0732. This exemption was granted due to the nature of the study, which involved anonymous survey data without any identifiable personal information.

## Results

A total of 230 individuals from 18 different departments across the US responded to the survey. Respondents included faculty from 18 different institutions across the country (Table 1), including individuals who held roles as residency core faculty, non-core faculty, and residency and department leadership. There was a wide range of clinical experience from 0-5 years (25%) to 21+ years (20%) (Table 2). Overall, the majority of faculty were satisfied with current grand rounds, as 68% of faculty rated their current grand rounds between a 7-10 out of 10 (Table 2).

**Table 1:** Practice location of respondents, with number and percentage of responses SUNY: State University of New YorK; UNC: University of North Carolina; UCSF: University of California San Francisco; N/A: Not Applicable

Institution	City and State	Number of Responses	% of Responses
Brown University	Providence, RI	31	14%
SUNY Upstate Medical Center	Syracuse, NY	27	12%
Staten Island University Hospital	Staten Island, NY	19	8%
UNC - Chapel Hill	Chapel Hill, NC	18	8%
Rutgers Health Community Medical Center	Toms River, NJ	14	6%
University of Maryland	Baltimore, MD	14	6%
Medical University of South Carolina	Charleston, SC	13	6%
Carl R Darnall Army Medical Center	Fort Cavazos, TX	12	5%
UCSF - Fresno	Fresno, CA	12	5%
Pennsylvania State University	Hershey, PA	9	4%
University of Central Florida	Orlando, FL	9	4%
University of South Alabama	Mobile, AL	9	4%
Prisma Health - Upstate	Greenville, SC	8	3%
Dignity Health - East Valley	Gilbert, AZ	7	3%
Eastern Virginia Medical School	Norfolk, VA	6	3%
Maimonides	Brooklyn, NY	6	3%
New York University - Long Island	Mineola, NY	6	3%
Metharlem Emergency Medicine	Bronx, NY	5	2%
Other	N/A	4	2%

**Table 2:** Description of respondents and current state of GR GR: Grand Rounds; PD: Program Director; APD: Assistant/Associate Program Director

Position	% of Responses	Years Post Residency	% of Responses	Number of GR Sessions Per Year	Offered % Responses	Preferred % Responses	Attended % Responses	Overall Satisfaction	% of Responses
Faculty member	35	0-5	25	0	1	1	6	1-2	3
Residency core faculty	29	6-10	26	1-4	31	17	53	3-4	9
Chair, vice chair, or medical director	16	11-15	14	5-8	23	27	26	5-6	19
PD or APD	15	16-20	16	9-12	26	34	8	7-8	47
Clerkship director	3	21+	20	13+	18	21	7	9-10	21

**Figure 1:**
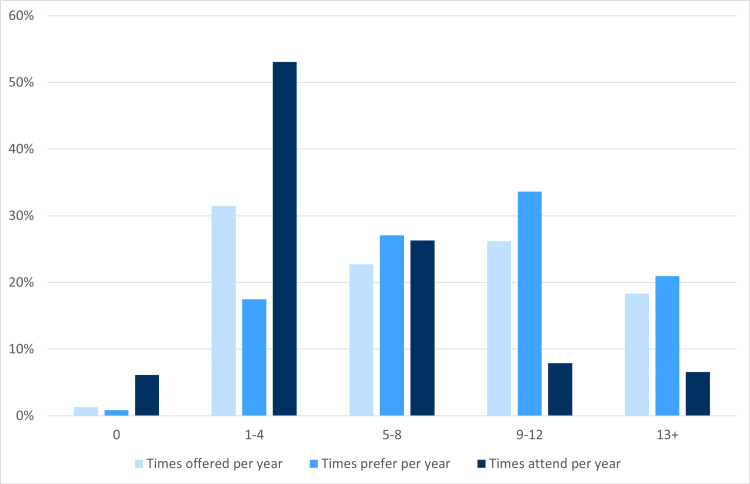
Number of times GR sessions are offered, the number of times respondents would prefer to have sessions offered, and the number of times respondents attend per year GR: Grand Rounds

The frequency at which grand rounds are offered was variable (from 0 times per year to 13+). There was also a wide range of the desired frequency of grand rounds sessions (Table 2 and Figure 1). The number of times grand rounds sessions are offered at respondents’ institutions is shown in Table 2 and Figure 1. Around 53% of faculty attended grand rounds 1-4 times per year, while most of the rest attended more frequently. However, a majority (55%) preferred that grand rounds be offered 9-13+ times per year. In the open-ended questions about attendance requirements, there was a high degree of variability, from no attendance requirement to a requirement to attend a certain percentage of sessions or hours per year.

Lectures were the most offered education format, cited by 207 (90%) participants, followed by hands-on sessions, 151 (66%), interactive workshops, 109 (47%), journal clubs, 106 (46%), and informal discussions, 57 (25%) (Table 3). A hybrid format (virtual + in-person) was the most desired delivery format as well as the most common delivery method for 68% and 76% of respondents, respectively (Table 3).

**Table 3:** Offered and desired formats of educational GR sessions GR: Grand Rounds

Format	% of Responses With Format	Modality	% With Offered Format	% Preferred Format
Lectures	90	Hybrid	76	68
Hands-on	66	In-person only	29	20
Interactive workshops	47	Recorded videos	10	23
Journal clubs	46	Virtual only	7	10
Informal conversations	25	Other	5	2
Other	4	-	-	-

**Figure 2:**
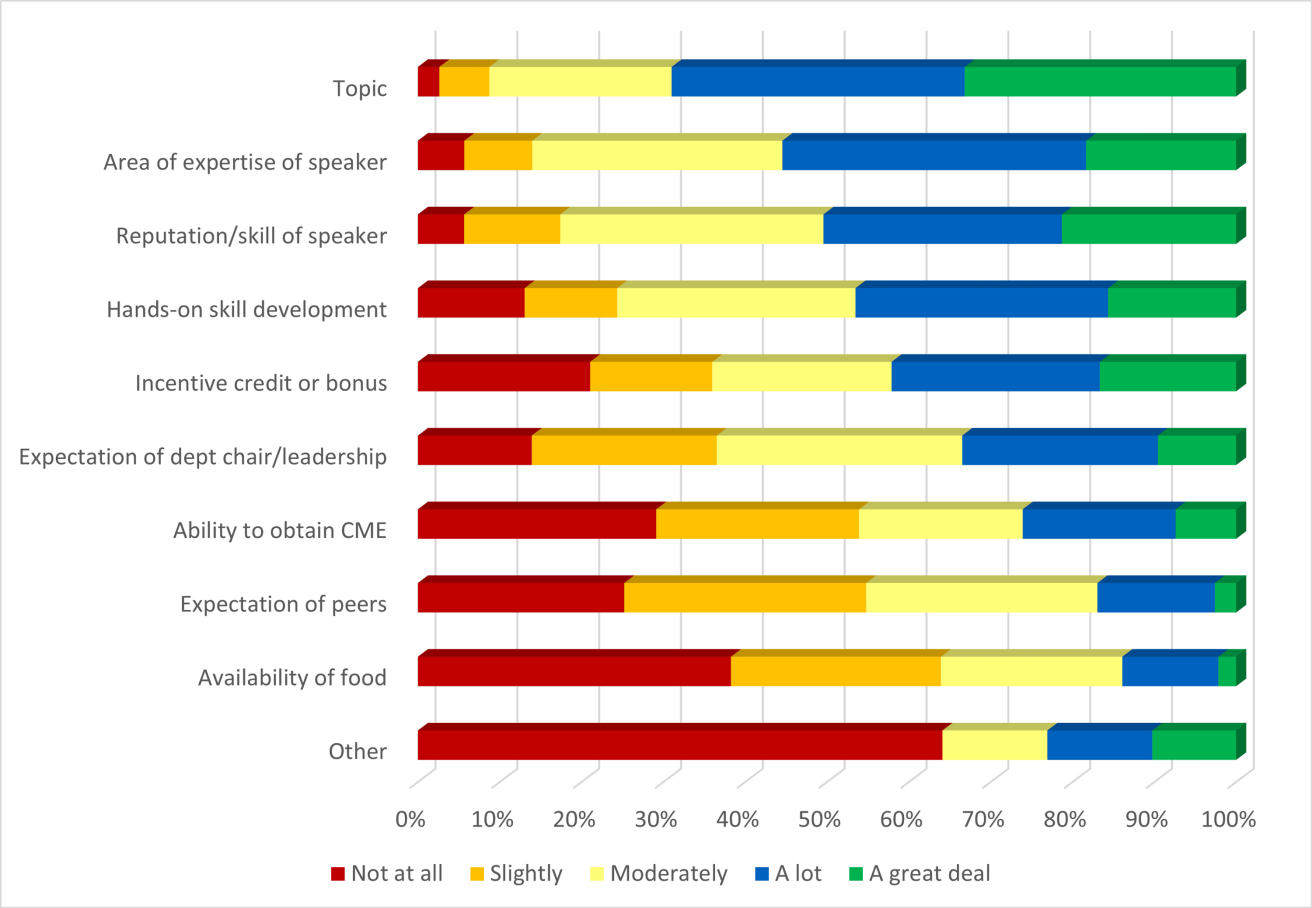
Motivation to attend GR sessions, sorted in descending order of the sum of “a lot” and “a great deal” responses GR: Grand Rounds; CME: Continuing Medical Education

We surveyed individuals on their motivation to attend sessions. Around 76% of respondents cited content topic as a very important motivator to attend sessions, followed by area of expertise of the speaker (42%), reputation/skill of the speaker (49%), incentive credit or bonus (38%), hands-on skill development opportunities (36%), and expectation of the department chair (22%) (Figure 2).

**Figure 3:**
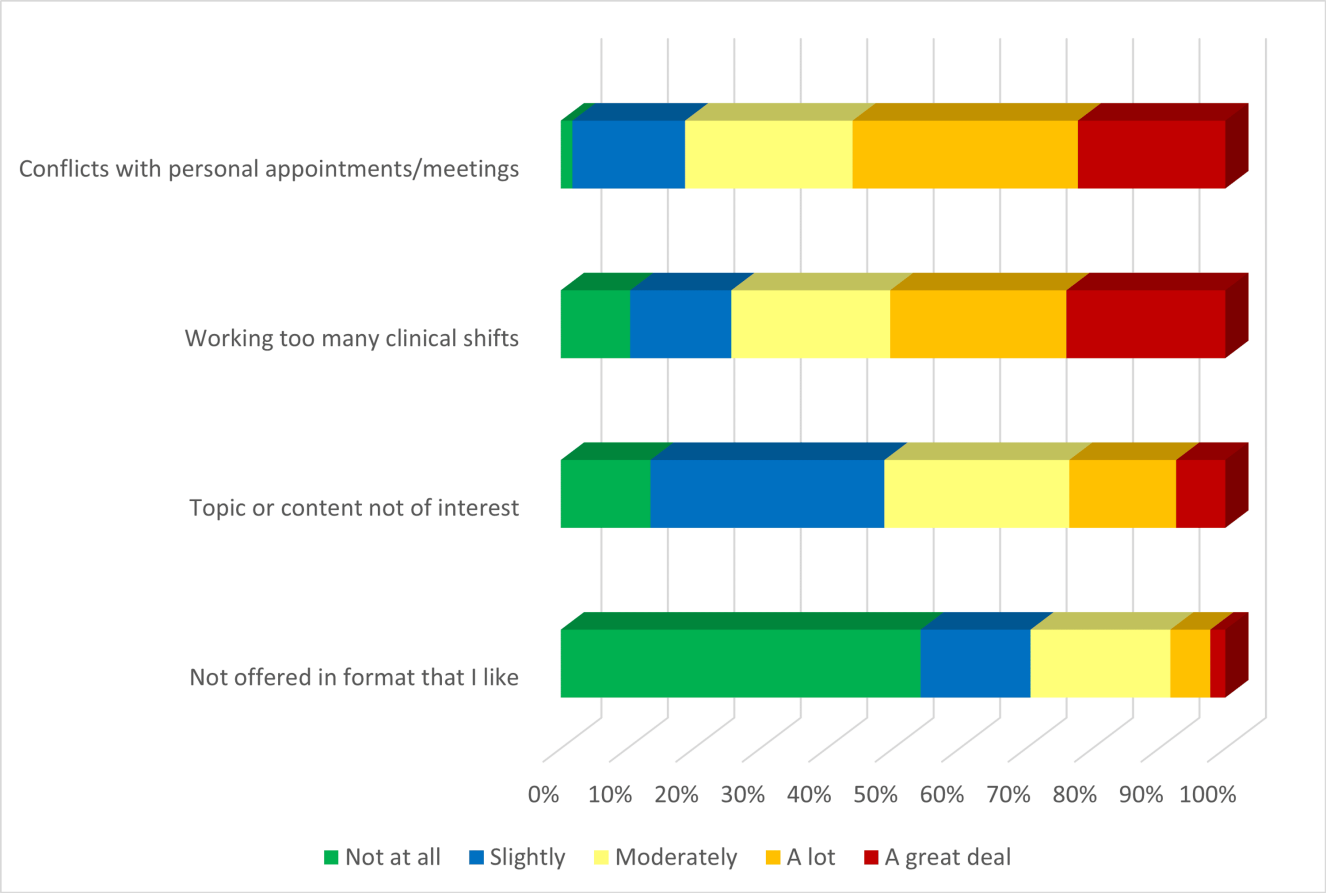
Barriers to attending GR sessions, sorted in descending order of the sum of “a lot” and “a great deal” responses GR: Grand Rounds

Given the demands of shift work, other academic meetings, and personal schedules, there are often barriers that arise to attending grand rounds sessions. Around 55% of faculty considered working too many clinical shifts a significant barrier to attending grand rounds. Additional major barriers were conflicts with personal meetings or appointments (Figure 3).

A thematic analysis of the open-ended questions found that over the past five years, faculty cited the following areas as the most impactful topical areas presented: procedural skills/hands-on training, feedback and education, cardiology/critical care, neonatal/pediatric care, and leadership/professional development. Faculty also noted that they would prefer more content on procedural skills/hands-on training, critical care/resuscitation, faculty development/teaching techniques, cardiology/electrocardiogram interpretation, and public health/systems of care over the coming year.

Faculty recommended the following changes that would improve their grand rounds sessions: more interactivity and hands-on training, incentives for attendance, and better scheduling and accessibility, such as hybrid, asynchronous, or virtual options to allow more flexibility.

Faculty also have many other options for obtaining training and CME credits. The most commonly used resources for continuing education cited by faculty included Emergency Medicine: Reviews and Perspectives (EM:RAP), podcasts, conferences (national/in-person/virtual), UpToDate, and journals.

## Discussion

Physicians are lifelong learners. To improve their skills and knowledge and learn new developments in the rapidly changing medical environment, they must participate in regular, high-quality education. This education is often delivered in part through departmental grand rounds sessions, which have long been a staple of medical education and academic discourse [1,4,5].

This study is the first to assess the motivation, barriers, and interests of EM faculty for their departmental grand rounds training. While prior work has addressed the format and evolution of grand rounds [4-6], few have examined the preferences and perspectives of EM faculty specifically [7,8].

We surveyed over 220 EM faculty members at 18 different academic institutions in the US to understand their needs, interests, and barriers to participation to help inform improvements in faculty-directed departmental educational offerings.

Faculty are the most motivated to attend sessions based on the content, the speaker’s area of expertise and reputation, as well as the ability to have hands-on skill development similar to findings in prior work that highlight the appeal of expert-led, skill-based curricula [3,8]. There are many barriers and competing priorities that keep faculty from attending, primarily their shifts and other meetings and responsibilities. Considering this, they prefer hybrid options to allow for more flexible attendance opportunities, consistent with recent studies exploring virtual and hybrid grand rounds formats in the wake of COVID-19 [9-14].

Finally, as organizers are selecting speakers, they should keep in mind the areas that the faculty most want to learn about or that are most memorable. In this survey, we brought to light the key areas in which faculty want more education. The topics tended to cluster around high acuity or procedural skills as well as teaching and professional development topics.

This study has several limitations. As a survey-based study, there may be a potential survey response bias. The focus of this study was academic medical faculty, which may limit its generalizability to non-academic sites. In addition, while we successfully surveyed over 200 faculty members from 18 institutions, there may be additional variability across the more than 200 academic medical centers in the US, which could limit generalizability. Future research can further hone the broad interests of EM faculty and assess the effectiveness of educational sessions through outcomes or quality improvements.

## Conclusions

Given the busy schedules of academic EM physicians and the necessity of regular continuing education, faculty grand rounds sessions provide an important opportunity to contribute to faculty development, engagement, and ultimately, high-quality patient care. Faculty will be more engaged and motivated to attend when the topics, speakers, format, and schedule optimally meet their needs and interests. This study can help guide faculty development efforts. We also hope it will serve as inspiration to survey faculty at a site to determine their needs and interests in order to better inform faculty development programming.
